# Trends in psychotropic medication across occupation types before and during the Covid-19 pandemic: a linked administrative data study

**DOI:** 10.1007/s00127-025-02909-0

**Published:** 2025-05-21

**Authors:** Finola Ferry, Lisa Kent, Michael Rosato, Emma Curran, Gerard Leavey

**Affiliations:** 1 Bamford Centre for Mental Health and Wellbeing, Ulster University, Coleraine, BT52 1SA Northern Ireland; 2 Administrative Data Research Centre Northern Ireland (ADRC-NI), Belfast, Northern Ireland; 3 School of Medicine, Dentistry and Biomedical Sciences, Centre for Public Health, Queens University Belfast, Belfast, BT12 6BA Northern Ireland

**Keywords:** COVID-19, Administrative data, Mental health, Occupation, Psychotropic medications

## Abstract

**Purpose:**

Using linked administrative data, this study provides the first longitudinal analysis of mental health among workers across occupational groups prior to and during the Covid-19 pandemic. Eleven years of data were analysed to examine whether the pandemic period coincided with changes in psychotropic medication for workers across broad occupational groupings.

**Methods:**

Data from Northern Ireland (NI) Enhanced Prescribing Database (EPD) was linked with NI Longitudinal Study (NILS) to examine trends in anti-depressants, anxiolytics and hypnotics (2011–2021) among NI workers (*N* = 200,004) across nine major occupation groups. Quarterly prescriptions were examined prior to and during pandemic restrictions (Q1-2011 to Q4-2019; and Q1-2020 to Q4-2021, respectively). Auto-regressive integrated moving average (ARIMA) models were trained to compare ‘forecasted’ and ‘observed’ rates during the pandemic period, stratified by occupational group.

**Results:**

Q2-2020 coincided with lower-than-expected receipt of anxiolytics and anti-depressants for several broad occupation types. Receipt of anxiolytic prescriptions among *managers*,* directors/senior officials* dropped below expected levels for the three quarters from Q3-2020 to Q1-2021. *Finally*, a notable increase in anti-depressants for a prolonged period was found among staff in caring/leisure and related professions, as well as higher rates of hypnotics in Q2-2021.

**Conclusion:**

Our study provides the first longitudinal examination of variation in mental health across occupation types prior to and during the Covid-19 pandemic, using available linked administrative data. Findings suggest that occupation type was an important pandemic-related stressor and point to potential higher risk occupations that could be the focus of targeted interventions in future pandemics.

**Supplementary Information:**

The online version contains supplementary material available at 10.1007/s00127-025-02909-0.

## Introduction

The COVID-19 pandemic in early 2020 created major challenges for daily working life for millions of workers, most experiencing fundamental but varying changes, depending on occupation types and roles. Frontline personnel such as police, healthcare and supermarket workers continued with in-person roles but faced adaptations to regular protocols, increased workloads, and higher risks of infection. There was a major shift towards remote working, with an increase in exclusive home working from 5.7% of the workforce in January/February 2020 to 43.1% in April 2020 [1]. Many workers availed of job retention schemes; were made redundant; or faced reduced working hours (and earnings) or precarious and insecure work patterns. One in four United Kingdom (UK) workers were furloughed at some point between March 2020 and June 2021, with those in hospitality, construction, and recreation among the most likely [2]. Redundancy rates were highest among those working in the administrative and support industry, while the transport industry experienced a four-fold increase in redundancy [3].

Numerous studies have found that the pandemic had an adverse effect on population mental health [4–7]. Concerns about financial difficulties related to the pandemic were commonly ranked above concerns about becoming unwell with Covid-19 [7], with uncertainty around job security having a particularly detrimental impact on mental health among workers [8]. However, data on actual and expected trends in psychotropic medication uptake from 2012 through the first year of the pandemic suggest that, aside from initial ‘stockpiling’ of medications in the early months of the pandemic, trends returned to expected levels thereafter [9]. While trends in psychotropic medication across various factors were considered in this study, variation in medication across occupation types prior to and during the pandemic has yet to be examined.

Evidence from recent global recessions and the wider literature on work and mental health highlight the effects of debt, financial strain, increased uncertainty, loss of structure, purpose and identity [10–14], and point to the greater vulnerability of lower income workers; those working in insecure and precarious jobs, and those working instressful jobs [15–17]. Just as the *economic impact* of the pandemic was not uniformly experienced across occupation types, it is likely that the *mental health impact* was also heterogeneous. The detrimental mental health impact of the pandemic on healthcare staff associated with long hours, pressured environments, exposure to trauma, moral dilemmas, lack of equipment and PPE among other stressors has been the focus of numerous studies [18–21]. Beyond studies of healthcare workers, however, little evidence has emerged examining other occupational groups. Furthermore, we found no longitudinal studies linked to the Covid-19 pandemic on mental health of workers that account for trends in mental health in preceding years, or studies that examine temporal trends *across occupation types*.

In preparation for future pandemics there is a need to better understand stress and wellbeing in different occupations. As far as we know, this study, based on administrative data, is the first longitudinal analysis of mental health among workers across occupational groups prior to and during the Covid-19 pandemic. Using individual level, linked administrative data, this study examines trends in quarterly rates of psychotropic prescriptions (prescriptions used to treat mental health conditions) among individuals in nine broad occupation types from quarter 1 (Q1) 2011 to Q4 2021. For each occupational group, we examined whether actual prescription rates during the pandemic period deviated from the forecasted or expected trend. Informed by the limited evidence base and heightened exposure to Covid-related stressors, we hypothesized that those working in caring professions would demonstrate higher than expected rates of psychotropic medication during the pandemic period. Beyond this, our analysis is largely exploratory.

## Materials and methods

### Data

This study is part of the Administrative Data Research (ADR) initiative, funded by the UK Economic and Social Research Council (ESRC) to develop the use of routinely collected administrative data for research purposes. Analysis for this study was based on linkage of data from the Northern Ireland Longitudinal Study (NILS) and NI Enhanced Prescribing Database (EPD). While the NILS is described elsewhere [22], briefly it is a 28% sample of the NI-Census enumerated population. The population of interest comprised all NILS members living in private households, in employment and enumerated at the 2011 Census. The NI EPD holds information on all medications dispensed in community pharmacies nationwide. The study was limited to those aged 18–54, capturing individuals who were still of working age by 2021. Individuals enumerated in the Census who died during the follow-up period (2011 to 2021) were excluded from analyses, providing a final core sample of 200,004 individuals. Census data was linked to contiguous quarterly (three-monthly) indicators of psychotropic medication prescriptions from the EPD from Q1-2011 to Q4-2021, held and provided by the Health and Social Care Business Services Organisation (HSC BSO). Data linkage was performed by the Northern Ireland Statistics and Research Agency (NISRA) Research Support Unit (RSU) and data were de-identified and pseudonymised prior to release to the research team. Participant consent was not required for this study. The data is classed as confidential: it is accessed from within a *secure setting*; by *accredited* researchers working under stringent protocols obviating disclosure problems. Ethical approval for this study was granted by Wales NHS Research Ethics Committee (REC), December 2021 (REC Reference: 21/AW/0362).

### Outcomes

Analysis focused on three psychotropic medication types, assumed to be proxy indicators of mental health conditions. Specifically, over the 44-quarter study period, data were extracted for any anti-depressant, anxiolytic, and hypnotic medications based on British National Formulary (BNF) classifications 4.1.1, 4.1.2 and 4.3 respectively. Anti-depressant medication included tricyclics (4.3.1); selective serotonin reuptake inhibitors (SSRIs) (4.3.2); and other anti-depressant drugs (4.3.3). Quarterly dichotomous indicators of receipt of these prescription types (0 = no, 1 = yes) were defined from Q1-2011 to Q4-2021.

### Explanatory factors

We examined, separately, trends in anti-depressant, anxiolytic and hypnotic prescriptions across nine broad occupational groups as defined by the Standard Occupational Classification (SOC) coding schedule, a hierarchically organised system with nine major groupings: 1) Managers, directors and senior officials; 2) Professional occupations; 3) Associate professional occupations; 4) Administrative and secretarial roles; 5) Skilled trades; 6) Caring, leisure and other service roles; 7) Sales and customer service occupations; 8) Process, plant and machine operatives; and 9) Elementary occupations. Classification of individuals across these occupation types represents their occupational status at the point of the 2011 NI Census. While more specific occupation types of interest could have been selected for analysis, we chose to analyse trends across broad groupings to profile the landscape in mental health prescriptions among the working population over the pandemic period; and gain an initial insight into the differential impact on workers in different types of jobs. The full occupational classification is outlined by the Office for National Statistics (ONS) [23].

Descriptive analysis also examined receipt of any psychotropic medication across a range of other individual and household characteristics (as recorded in Census 2011): sex (male, female); age-group (16–24,25–34,35–44,45–54 years); marital status (married/civil partnership, single, separated/divorced/widowed/dissolved); household car access (none, one, two or more); education level (degree, intermediate, no qualifications); settlement (urban, intermediate, rural); weekly hours worked (15 or less, 16–30, 31–48, 49 or more); weekly caregiving responsibilities (none, 1–19 h, 20–49, 50 or more); and any self-reported chronic health condition (excluding a mental health condition) – these included deafness, blindness, mobility difficulty, long-term pain, breathing difficulty, chronic illness, learning difficulty, memory loss, communication difficulty, and any ‘other disorder’.

### Analysis

#### Descriptive analyses

Data pre-processing and descriptive analyses were performed in Stata version 18.0 [24] and R Statistical analyses were performed using R packages including *tidyverse* and forecast [25, 26]. Individuals were identified as being in receipt of medication if they were dispensed one or more prescriptions in that quarter. Descriptive analysis examined the proportion of workers in receipt of each prescription type in each quarter across major SOC and other individual and household level characteristics.

#### Pre-existing trends quarter 1-2011 to quarter 4-2021

The number of patients receiving prescriptions each quarter was calculated, and, using the quarterly denominator (which was consistently 200,004, given the exclusion of all deaths over the study period), expressed as a percentage of the eligible population. Line graphs were generated to visualise trends over time for each medication.

#### Impact of restrictions on uptake of medications

In the absence of widely accepted guidelines for the reporting of time-series analyses, this paper adheres to the recommendations set out by Jandoc and colleagues [27]. Data for each medication type were separated into pre-restrictions (Q1-2011 to Q4-2019) and during restrictions (Q1-2020 to Q4-2021). A central problem with scanners used to record prescriptions meant that the recorded data on prescriptions dispensed in 2017 and 2018 were artificially lowered by approximately 10% compared with previous years. Previous work by Maguire et al. [9] ascertained that ARIMA models trained on time series data with 2017–2018 removed performed best. This was confirmed in the current study by comparing the corrected Akaike information criterion (AICc) associated with a range of models developed using quarterly time series of individuals receiving antidepressants: (a) exponential smoothing model with 2017/18 data present (AICc: 563.55), (b) ARIMA with 2017/18 data present (AICc: 466.72), (c) exponential smoothing with 2017/18 treated as missing (AICc: 502.72), and (d) ARIMA with 2017/18 treated as missing (AICc: 304.33).

As a result, ARIMA models developed on data with 2017/18 values removed were used for the remaining analyses. Training data comprised time series of ‘counts of individuals dispensed prescriptions’ as opposed to quarterly counts of ‘prescriptions’, and was repeated for each medication type (antidepressants, anxiolytics, and hypnotics) for the full population and stratified by each occupation classification. Each model was trained using the *auto.arima()* function. The algorithm was permitted to iteratively attempt to fit on differenced data (to remove trend) and first seasonal difference (to remove seasonal trend) and automatically choose the remaining parameters that resulted in the best fit. A transfer function (i.e. shape of the impact after initial introduction of intervention) was not added to the ARIMA model as restrictions over the time period studied were subject to change.

The trained models were then used to forecast medication uptake for each quarter in the time frame 2020 Q1 to 2021 Q4. The expected (i.e. forecast) quarterly values and upper and lower 80% and 95% confidence limits were extracted and plotted against actual quarterly values. This allowed identification of actual quarterly values that lie outside of the confidence limits for the expected values. Observed to expected ratios (and 95% confidence intervals) were also calculated. This was repeated for each medication type and stratified by SOC.

## Results

The characteristics of individuals in the study cohort and the proportions recorded as having receipt of the three classes of psychotropic medication at any point over the study period are presented in Table 1. Overall, respectively 45.5%, 25.8% and 18.3% of workers were in receipt of antidepressant, anxiolytic and hypnotic medication between 2011 and 2021.

With respect to medication uptake across occupation types, those working in *caring*,* leisure and other service* roles recorded the highest rates of receipt of each of the three medication classes over the eleven-year period (58.7% for antidepressants, 35.2% for anxiolytics and 24.0% for hypnotics). Those in *sales/customer service* roles and in *elementary* occupations also recorded consistently high rates of uptake across all three classes of medication. Uptake was lowest among those working in *professional* occupations (35.5% for antidepressants, 20.2% for anxiolytics and 13.6% for hypnotics).

A higher proportion of females were in receipt of each of the three prescription classes, with over half of females (54.7%) receiving anti-depressant medication, compared to 35.6% of males. Uptake of medication generally increased with age, was higher among those who were previously married, as well as those with no household car access or educational qualifications. Receipt of medication was lower among those living in rural areas, while for people in urban and intermediate areas the rates were similar. Uptake of all three classes of psychotropic medication decreased as the number of working hours increased, while workers with intense caregiving responsibilities (fifty hours of more per week) and those with other self-reported chronic conditions had higher uptake of medication.

### Psychotropic medication trends among workers 2011–2021

**Fig. 1:**
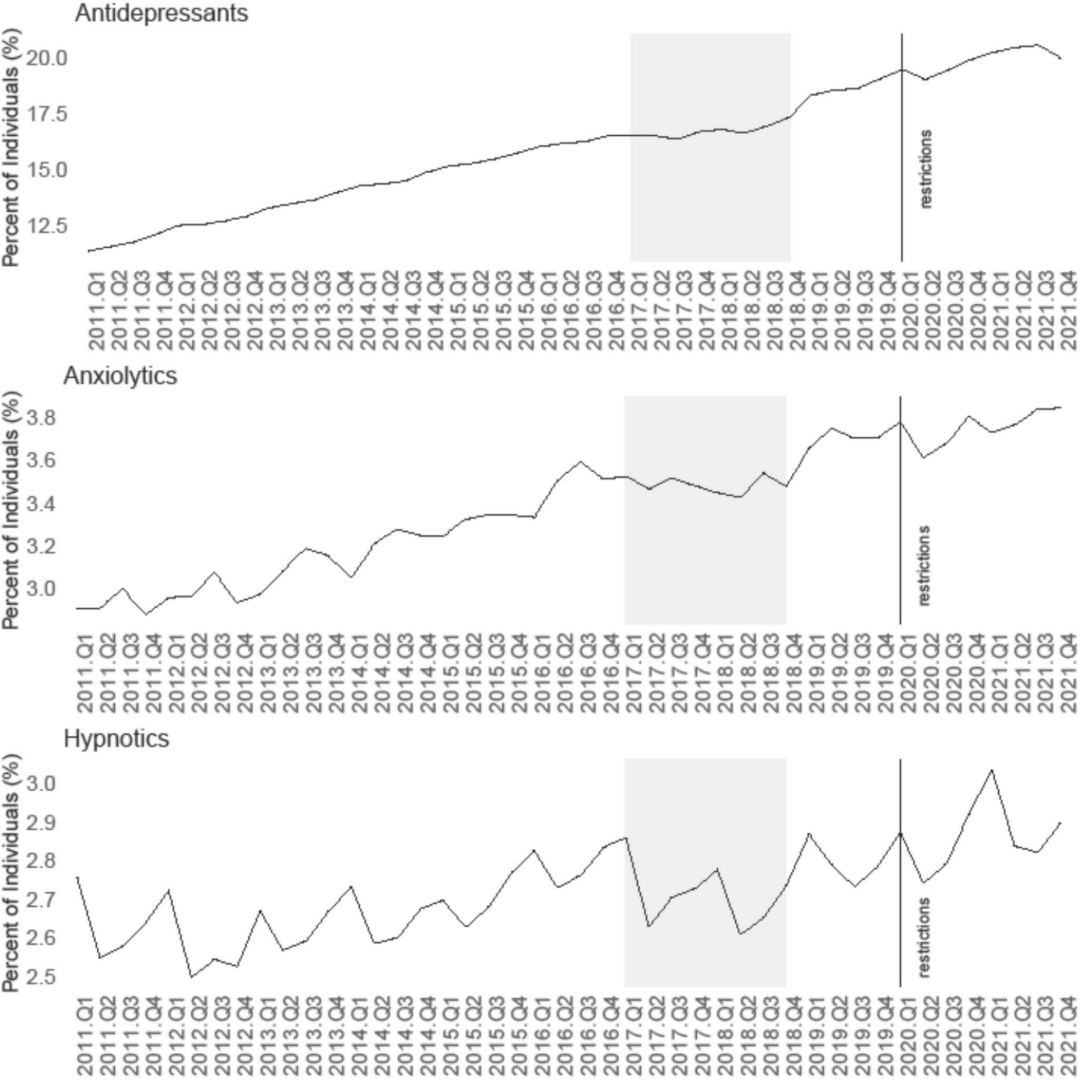
Percentage of workers (*N* = 200,004) in receipt of ‘any anti-depressant’, ‘any anxiolytic’ and ‘any hypnotic’ medication each quarter from Quarter 1 2011 to Quarter 4 2021 (vertical line depicts onset of pandemic in Quarter 1 2020 and shaded areas 2017–2018 depict years affected by prescription scanning problem)

Receipt of anxiolytic and antidepressant medications among workers followed a steady upward trend across the whole follow-up period (Fig. 1). While receipt of antidepressants displays a relatively smooth upward trend, seasonal fluctuations are apparent in the uptake of anxiolytics. While there was a notable downward spike at Q2-2020 in anxiolytic uptake, it appears that the overall upward trajectory for both anxiolytics and antidepressants maintained during the pandemic period. The trend for hypnotic medication from 2011 to 2021 (Fig. 1) shows that uptake fluctuated around a relatively flat level, with a consistent pattern (higher in Q4/Q1 and lower in Q2) over the whole period. This trend continued throughout the pandemic period; however, the overall hypnotics uptake rate appears higher following the onset of restrictions, with the upward spike at Q2-2020 more pronounced than in previous years.

### Impact of restrictions on receipt of medication across occupation types

ARIMA models for trend were trained by major SOC for antidepressants, anxiolytics, and hypnotics separately. Overall (Supplementary material Fig. 1), observed trends in all three categories of psychotropic medication fell within expected limits, with a few exceptions: receipt of anti-depressants was lower than forecast in Q2-2020 and Q4-2021; receipt of anxiolytics was also lower than expected in Q2-2020; while receipt of hypnotics was elevated in Q1-2021 (although on the margins of significance).

**Fig. 2:**
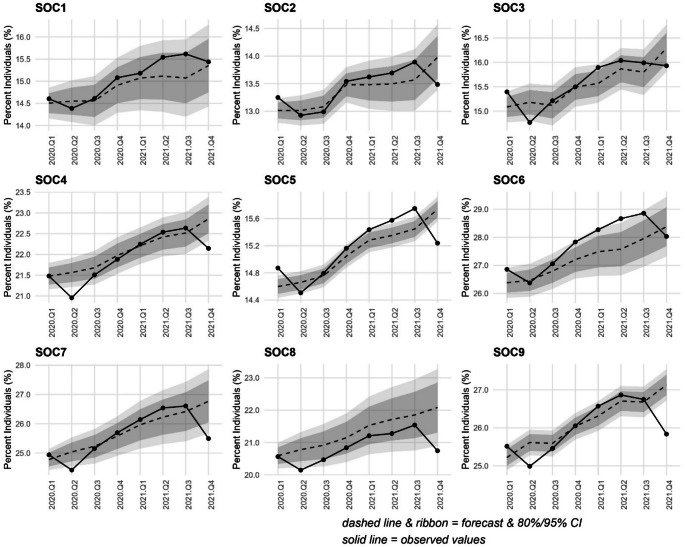
Auto regressive integrated moving average (ARIMA) illustrating forecast versus actual values of uptake of anti-depressant medications during the first 8 quarters of the COVID-19 pandemic (Quarter 1 2020 to Quarter 4 2021) among NILS members by standard occupational classification (SOC). Dark grey band denotes the 80% confidence interval and the light grey band the 95% confidence interval. SOC1. Managers, directors and senior officials; SOC2. Professional occupations; SOC3. Associate professional occupations; SOC4. Administrative and secretarial occupations; SOC5. Skilled trades occupations; SOC6. Caring, leisure and other service occupations; SOC7. Sales and customer service occupations; SOC8. Process, plant and machine operatives; SOC9. Elementary occupations

**Fig. 3:**
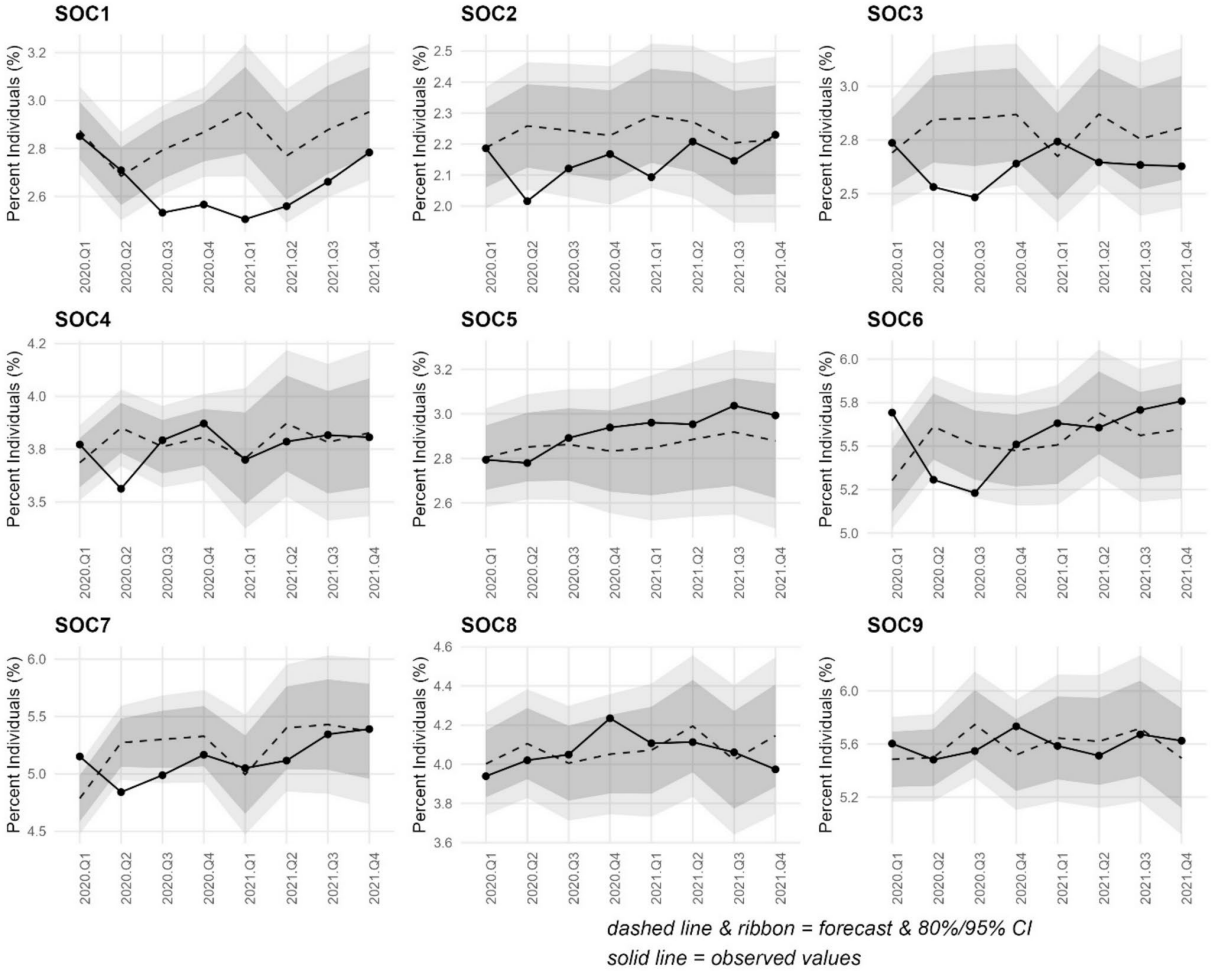
Auto regressive integrated moving average (ARIMA) illustrating forecast versus actual values of uptake of anxiolytic medications during the first 8 quarters of the COVID-19 pandemic (Quarter 1 2020 to Quarter 4 2021) among NILS members by standard occupational classification (SOC). Dark grey band denotes the 80% confidence interval and the light grey band the 95% confidence interval. SOC1. Managers, directors and senior officials; SOC2. Professional occupations; SOC3. Associate professional occupations; SOC4. Administrative and secretarial occupations; SOC5. Skilled trades occupations; SOC6. Caring, leisure and other service occupations; SOC7. Sales and customer service occupations; SOC8. Process, plant and machine operatives; SOC9. Elementary occupations

**Fig. 4:**
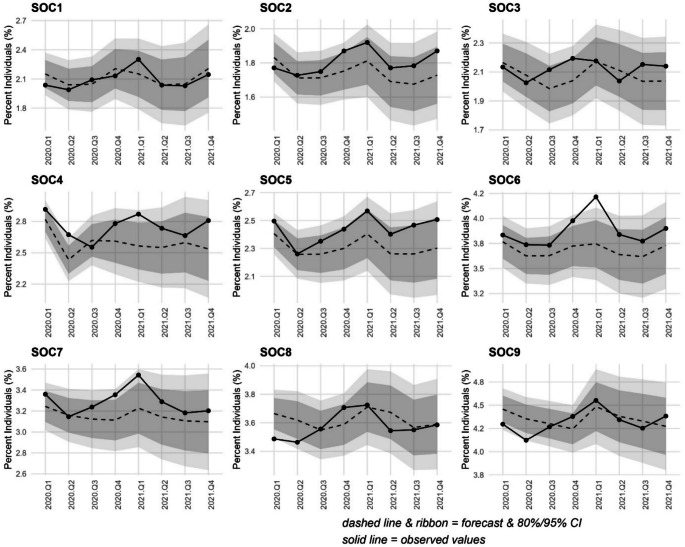
Auto regressive integrated moving average (ARIMA) illustrating forecast versus actual values of uptake of hypnotic medications during the first 8 quarters of the COVID-19 pandemic (Quarter 1 2020 to Quarter 4 2021) among NILS members by standard occupational classification (SOC). Dark grey band denotes the 80% confidence interval and the light grey band the 95% confidence interval. SOC1. Managers, directors and senior officials; SOC2. Professional occupations; SOC3. Associate professional occupations; SOC4. Administrative and secretarial occupations; SOC5. Skilled trades occupations; SOC6. Caring, leisure and other service occupations; SOC7. Sales and customer service occupations; SOC8. Process, plant and machine operatives; SOC9. Elementary occupations

Figures 2, 3 and 4 provide a more detailed illustration of observed versus expected trends across the nine broad occupation types. An alternative presentation of these findings (observed to expected ratios) as well as summary tables are also provided in Supplementary Material. ARIMA models comparing observed and forecasted trends in antidepressant medication (Fig. 2) show that receipt largely fell within the expected limits for *managers*,* directors/senior officials* (SOC1) and *professional* occupations (SOC2), although higher rates *coincide* with the onset of restrictions in Q1 for the latter. For those working in *caring*,* leisure/other service* occupations (SOC6) higher rates were observed for the twelve months from Q4-2020 to Q3-2021 (although for some quarters these differences were on the margins of significance). Receipt of antidepressant medication for Q2- 2020 was lower than expected for those working in *associate professional/technical (SOC3)*, *administrative/secretarial (SOC4)*, *sales/customer service (SOC7)*, *process*,* plant and machine (SOC8)* and *elementary* occupations (SOC9). Finally, a sharp downward shift in receipt of antidepressants occurred in Q4-2021, with lower-than-expected rates among *administrative/secretarial* occupations (SOC4), *skilled trades (SOC5)*, *sales/customer service (SOC7)*, *process plant and machine operatives* (SOC 8) and, most notably, *elementary* occupations (SOC9).

Turning to comparison of expected and forecasted values for anxiolytic medication receipt (Fig. 3); with some exceptions, uptake fell within the bounds of expected values for workers in most broad occupational groupings. However, uptake among *managers*,* directors/senior officials* fell below expected levels for a prolonged period from Q3-2020 to Q1-2021 inclusive. Mirroring findings in relation to anti-depressants, uptake in Q2-2020 was also lower than expected for those *professional*; *associate professional/technical*’ roles (for Q2-Q3 2020), *administrative/secretarial*, *caring*,* leisure/other service*, and *sales/customer service* occupations.

For hypnotics, except for workers in *caring/leisure/other service* roles, uptake of medication stayed within the expected limits throughout the entire study period (Fig. 4) - for this group receipt of hypnotics was significantly higher than forecast in Q2-2021, before returning to expected rates thereafter.

### Sensitivity analyses

The current study included all workers employed at the point of the 2011 Census regardless of their weekly working hours. To explore potential selection bias of inclusion of part-time workers, we ran ARIMA models based full-time workers only (Supplementary Material Figs. 5–8). At population level, there is broad agreement between the two cohorts (main cohort including full-time and part-time workers (FT/PT cohort) and full-time only cohort (FT cohort)). Each of the medications, however, showed a lower rate of dispensation when we restrict to FT workers. This remains true across all occupation types for antidepressants and anxiolytics, and for most occupation types for hypnotics. SOC6 (‘caring personal services’) sees an increased rate of hypnotics when we restrict to FT workers and may reflect the strain of long and unsociable hours in these occupations during the pandemic and associated increase in sleep disorders.

In terms of observed versus expected trends, there are a few occupation groups showing differences between the main analysis and sub-analysis. These differences are fully summarized in Supplementary Material.

**Table 1:** Socio-demographic characteristics and prevalence of hypnotics, anxiolytics and anti-depressants at any point between 2011 and 2021 among NILS eligible sample of workers aged 18–54

	Total eligible NILS population (*N* = 200,004)	Any anti-depressant medication 2011–2021, *n* (row %) (*n* = 90,978, 45.5%)	Any anxiolytic medication 2011–2021, *n* (row %) (*n* = 51,635, 25.8%)	Any hypnotic medication 2011–2021, *n* (row %) (*n* = 36,603, 18.3%)
Sex				
Male	96,420 (48.2)	34,298 (35.6)	18,437 (19.1)	14,062 (14.6)
Female	103,584 (51.8)	56,680 (54.7)	33,198 (32.1)	22,541 (21.8)
Age-group				
16–24	22,463 (11.7)	8,888 (37.9)	4,627 (19.7)	3,221 (13.7)
25–34	56,430 (28.2)	22,200 (39.3)	13,298 (23.6)	8,524 (15.11)
35–44	60,192 (30.1)	27,535 (45.8)	16,350 (27.2)	11,570 (19.2)
45–54	59,919 (30.0)	29,124 (48.6)	17,360 (29.0)	13,288 (22.2)
Marital Status				
Married/civil partnership	101,879 (50.9)	44,650 (43.83)	25,224 (24.8)	16,980 (16.7)
Single	77,191 (38.6)	33,149 (42.9)	18,421 (23.9)	13,158 (17.1)
Separated/divorced/widowed/dissolved	20,934 (10.5)	13,179 (63.0)	7,990 (38.2)	6,465 (30.9)
Major SOC				
Managers, directors and senior officials	14.729 (7.4)	5,770 (39.2)	3,163 (21.5)	2,306 (15.7)
Professional Occupations	32,241 (16.1)	11,448 (35.5)	6,509 (20.2)	4,390 (13.6)
Associate Professional/Technical	16,590 (8.3)	6,610 (39.8)	3,664 (22.1)	2,581 (15.6)
Admin and Secretarial	29,162 (14.6)	14,543 (49.9)	8,155 (28.0)	5,614 (19.3)
Skilled Trades	27,596 (13.8)	10,396 (37.7)	5,657 (20.5)	4,055 (14.7)
Caring, Leisure and other Service	19,692 (9.9)	11,549 (58.7)	6,927 (35.2)	4,723 (24.0)
Sales and Customer Service	19,646 (9.8)	10,761 (54.8)	6,103 (31.1)	4,248 (21.6)
Process, Plant and Machine Operatives	17,237 (8.6)	7,725 (44.8)	4,386 (25.5)	3,386 (19.6)
Elementary Occupations	23,111 (11.6)	12,176 (52.7)	7,071 (30.6)	5,300 (22.9)
Household car access				
None	22,142 (11.1)	13,306 (60.1)	8,004 (36.2)	6,396 (28.9)
One	65,803 (32.9)	33,113 (50.3)	18,742 (28.5)	13,878 (21.1)
2 or more	112,06 (56.0)	44,559 (39.8)	24,889 (22.2)	16,329 (14.6)
Education level				
Degree	71,458 (35.7)	26,192 (36.7)	14,554 (20.4)	9,924 (13.9)
Intermediate	100,574 (50.3)	48,602 (48.3)	27,218 (27.1)	19,089 (18.9)
No qualifications	27,972 (14.0)	16,184 (57.9)	9,863 (35.3)	7,590 (27.1)
Settlement				
Urban	74,548 (37.3)	35,377 (47.5)	20,331 (27.3)	14,731 (19.8)
Intermediate	68,207 (34.1)	32,664 (47.9)	18,447 (27.1)	13,445 (19.7)
Rural	57,249 (28.6)	22,937 (40.1)	12,857 (22.5)	8,427 (14.7)
Weekly hours worked				
15 or less	11,515 (5.8)	6,327 (55.0)	3,653 (31.7)	2,517 (21.9)
16–30	43,315 (21.7)	24,162 (55.8)	14,163 (32.7)	9,692 (22.4)
31–48	127,530 (63.8)	54,331 (42.6)	30,431 (23.9)	21,772 (17.1)
49 or more	17,644 (8.8)	6,158 (34.9)	3,388 (19.2)	2,622 (14.9)
Weekly caregiving hours				
none recorded	168,575 (84.3)	74,902 (44.4)	42,226 (25.1)	19,851 (17.7)
1–19	19,595 (9.8)	9,311 (47.5)	5,408 (27.6)	3,812 (19.5)
20–49	5,114 (2.6)	2,772 (54.2)	1,623 (31.7)	1,197 (23.41)
50 or more	6,720 (3.4)	3,993 (59.4)	2,378 (35.4)	1,743 (25.94)
Any other chronic condition				
No	161,645 (80.8)	66,941 (41.4)	37,236 (23.0)	25,663 (15.9)
Yes	38,359 (19.2)	24,037 (62.7)	14,339 (37.5)	10,940 (28.5)

## Discussion

Based on comparison of expected and actual trends in receipt of psychotropic medication, we examined changes in mental health during the pandemic period among workers in NI across broad occupation types. Trends in anti-depressant and anxiolytic medication among workers followed an upward trajectory from 2011 until the end of 2021, reflecting trends in mental health prescriptions reported elsewhere [9].

We found that receipt of all three sub-categories of mental health prescriptions fell within the expected limits for most occupation types (Q1-2020 to Q4-2021). However, occupation type may explain variation in mental health experienced among workers over the course of the pandemic.

*First*, Q2-2020 coincided with lower-than-expected receipt of anxiolytics and anti-depressants for several broad occupation types, including *associate professional technical* occupations; *sales/customer service* occupations; *administrative/secretarial* occupations, *professional* occupations (for anxiolytics), *process*,* plant/machine operatives* (for anti-depressants), and *elementary* occupations (for anti-depressants). *Second*, receipt of anxiolytic prescriptions among *managers*,* directors/senior officials* dropped below expected levels for the three quarters from Q3-2020 to Q1-2021. *Third*, while psychotropic prescriptions appear to have declined for some occupational groups during the pandemic, a notable increase in anti-depressant medication for a prolonged period was found among staff in caring/leisure and related professions, as well as higher rates of hypnotic prescriptions in Q2-2021.

Boden and colleagues [28] developed a population mental health framework as a means of understanding and addressing the mental health impact of the Covid-19 pandemic. Alongside social risk factors and clinical vulnerabilities, the framework considers the influence of pandemic-related stressors such as occupation type in increasing the risk of negative mental health outcomes. Various studies highlight adverse mental health effects associated with the pandemic among specific occupation types (healthcare workers [29–31]; hospitality workers [32]; and teachers [33]. However, there have been no population-level studies on occupation type as a pandemic-related risk factor. Nonetheless, our finding of limited impact of the pandemic on mental health prescription trends across most occupation types is in keeping with findings reported in a systematic review and meta-analysis, which found that there were no significant changes in general mental health or anxiety symptoms across general population studies that had examined the impact of the pandemic [34] and aligns with the idea that there was a level of *mental health resilience* during the pandemic reported elsewhere [35].

The diverse ‘caring personal service’ occupational classification includes nursing auxiliaries and assistants, ambulance staff (excluding paramedics) and care-workers, which constitute a substantial proportion. Our previous analysis on mental health prescriptions around the time of the 2011 Census found that workers in this broad occupational group had the highest rates of prescription receipt across all occupations, controlling for a wide range of socio-demographic and socio-economic characteristics [36]. In the current study, higher than expected rates of anti-depressant use for prolonged periods may reflect increased exposure of these ‘public-facing’ workers to numerous occupational stressors such as increased risk of Covid-19 infection, long working hours, stressful working environments, burnout, and moral dilemmas. Our findings are consistent with evidence from previous studies, which highlight healthcare workers as a high-risk group in terms of the adverse mental health effects of the pandemic [30, 37]. It is important to consider, however, that these workers may have specific knowledge of navigating health systems in flux or have access to care through employer health systems and prescribing, which may have had a bearing on prescribing patterns during the pandemic period. Higher hypnotics usage in Q2-2021 among other public-facing occupation types such as sales/customer service occupations coincided with significant reopening of society in the UK, which may reflect increased stress among returning workers [38],.

An explanation for lower-than-expected levels of anti-depressant and anxiolytic prescriptions among workers across several broad occupational groupings is more perplexing. It may reflect the wellbeing benefits associated with the switch to working from home [39], or increased family time and the strengthening of family relationships [40]. These themes however have not been evidenced in a robust manor in representative studies or indeed across occupation types. We also cannot determine whether this points to improved mental health or evidence of unmet need due to limited access to GP services. Our findings, however, suggest an expected balancing of the trend following the initial ‘stockpiling’ of psychotropic medication in NI at the very beginning or the pandemic [9]. Why anxiolytic medication among managers/directors/senior officials should decrease during the second half of 2020 and early months of 2021 is unclear, but perhaps homeworking resulted in more personal autonomy and reduced exposure to environmental stressors.

### Limitations

Study findings should be interpreted with a number of limitations in mind. First, given that the Census is the only available resource providing population-level data on occupation type, the occupation data used in this study was based on information collected at the 2011 Census. Analysis could not account for occupational mobility and therefore it cannot be assumed that individuals remained within a given SOC for the duration of the study period. We acknowledge the unavailability of longitudinal data on occupational group and employment transitions as a key limitation. Second, to provide an overall picture of the NI working population, this study focused on broad occupational categories, which encompass, in some cases, disparate jobs with potentially varying mental health outcomes prior to and during the pandemic period. A more fine-grained analysis of occupation types in future follow-up work would greatly enhance our understanding of the role that jobs play in pandemic-related health outcomes. Third, we acknowledge the general challenges associated with linking multiple administrative datasets from a variety of data providers, resulting in important aspects of the linkage process, which impact on the reliability of the final dataset, being obscured from those analysing and interpreting the linked data [41]. Fourth, we also acknowledge the risk of selection bias associated with the inclusion of PT alongside FT workers in our analysis, which have been documented in other studies [42–44]. Specifically, PT workers may have more underlying health conditions, including mental health disorders, and therefore more likely to use the prescriptions being studied. This was explored through sensitivity analyses which revealed some differences between full cohort versus the FT workers only cohort for certain occupation types. While our study is largely exploratory, with the focus being occupational differences, we recommend that future work accounts for potential confounding factors, including working hours and other health conditions. Fifth, while NI as part of the United Kingdom (UK) followed similar pandemic-related rules and protocols to other UK regions, a cautious approach should be taken to generalizing findings to other regions or populations, given varying approaches and policies in response to the pandemic. A final limitation – in the absence of details of treatment for any mental ill-health conditions this study used quarterly indicators of psychotropic medication as a proxy for mental ill-health. However, our findings of increasing rates over a decade coincide with increased prevalence of mental disorders and mental health care utilization reported elsewhere [45–47].].

## Conclusion

Our study provides the first population-wide examination of variation in mental health outcomes across occupation types prior to and during the Covid-19 pandemic, using available linked administrative data. It extends previous NI-based work on pandemic-related mental health outcomes, using more contemporaneous data that spans the duration of the pandemic through to the end of 2021, while incorporating pre-pandemic trends in analyses models. As outlined by Boden et al. [33], our findings suggest that occupation type was an important pandemic-related stressor and point to potential higher risk occupation types that could be the focus of targeted interventions in future pandemics. We recommend that future research in this area exploits the availability of more detailed three or four-digit standard occupational classification to unlock the potential of linked administrative data in its ability to inform targeted public policy around work and mental health. Approaches and strategies to support worker wellbeing during a pandemic will vary by industry, organization type and needs of the workforce [48]. Further, more fine-grained research in this area has the potential to inform the development of occupation specific mental health at work policies and strategies, such as reasonable adjustments, signposting and other occupational health support services, supportive leave policies, remote working, and other work arrangements that will support vulnerable workers and improve mental health resilience during public health emergencies.

## Electronic supplementary material

Below is the link to the electronic supplementary material.

Supplementary Material 1

## Data Availability

The linked administrative data that support the findings are safe-guarded and only available to members of the research team. Syntax files developed to produce findings reported in this study are available on request from the corresponding author.
