# Supplementary material for: Trends in psychotropic medication across occupation types before and during the Covid-19 pandemic: a linked administrative data study
Source: Soc Psychiatry Psychiatr Epidemiol. 2025 May 21;60(10):2311–23. doi: 10.1007/s00127-025-02909-0 (PMC12449321; doi:10.1007/s00127-025-02909-0)

Supplementary Material

Figure 1 Auto regressive integrated moving average (ARIMA) illustrating forecast versus actual values of uptake of psychotropic medications during the first 8 quarters of the COVID-19 pandemic (Quarter 1 2020 to Quarter 4 2021) among all NILS members in employment (includes fulltime and part-time workers) at the point of the 2011 Census. Dark grey band denotes the 80% confidence interval and the light grey band the 95% confidence interval


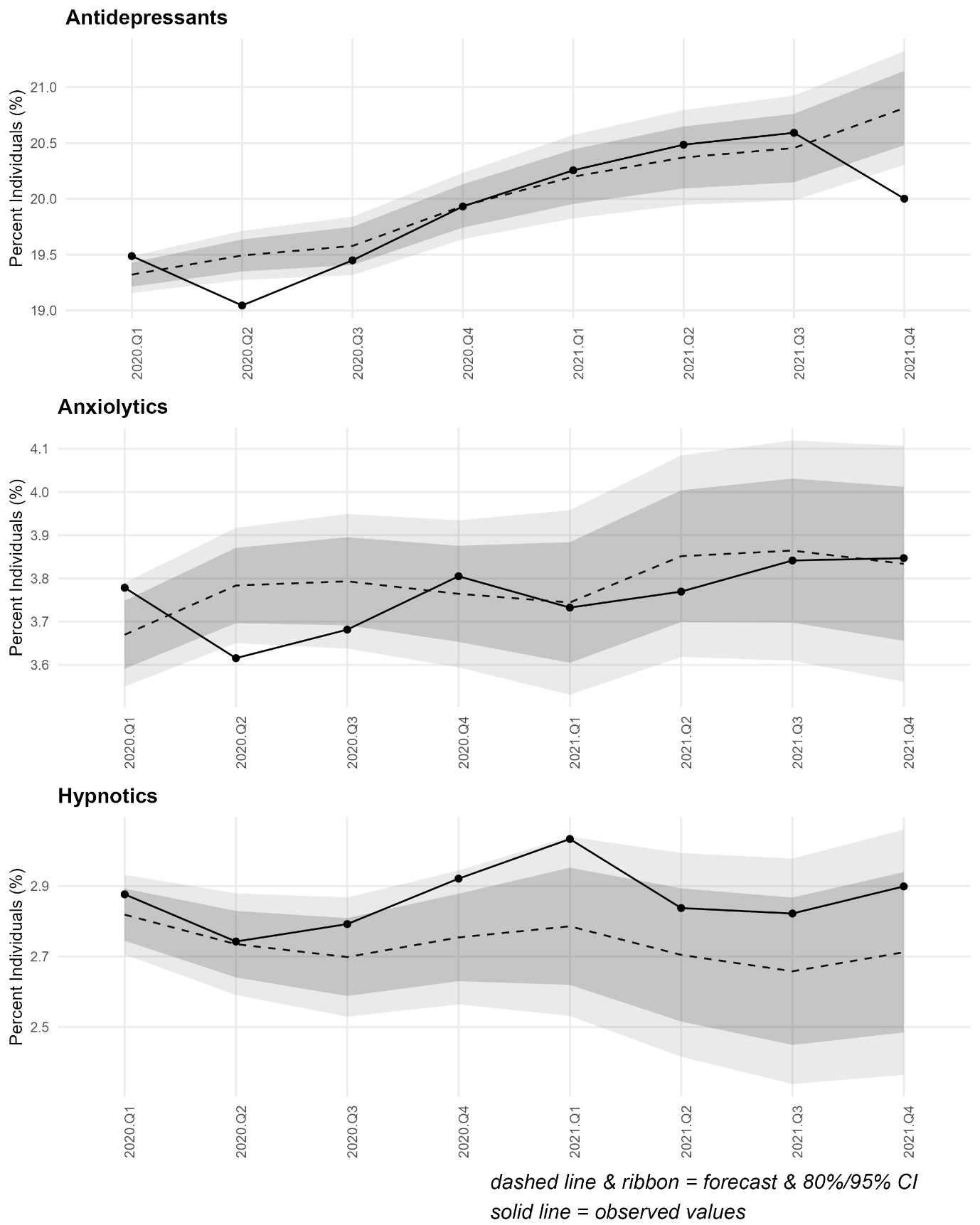


Table 1 Summary of findings from ARIMA models (based on full cohort) comparing expected and observed antidepressant prescription rates

| ↓ = significantly lower than expected; ↑ = significantly higher than expected | | | | | | | | |
| --- | --- | --- | --- | --- | --- | --- | --- | --- |
|  | 2020.Q1 | 2020.Q2 | 2020.Q3 | 2020.Q4 | 2021.Q1 | 2021.Q2 | 2021.Q3 | 2021.Q4 |
| SOC1 |  |  |  |  |  |  |  |  |
| SOC2 | ↑ |  |  |  |  |  |  |  |
| SOC3 |  | ↓ |  |  |  |  |  |  |
| SOC4 |  | ↓ |  |  |  |  |  | ↓ |
| SOC5 | ↑ |  |  |  |  | ↑ | ↑ | ↓ |
| SOC6 |  |  |  |  |  | ↑ |  |  |
| SOC7 |  | ↓ |  |  |  |  |  | ↓ |
| SOC8 |  | ↓ |  |  |  |  |  | ↓ |
| SOC9 |  | ↓ |  |  |  |  |  | ↓ |

Table 2 Summary of findings from ARIMA models (based on full cohort) comparing expected and observed anxiolytic rates

| ↓ = significantly lower than expected; ↑ = significantly higher than expected | | | | | | | | |
| --- | --- | --- | --- | --- | --- | --- | --- | --- |
|  | 2020.Q1 | 2020.Q2 | 2020.Q3 | 2020.Q4 | 2021.Q1 | 2021.Q2 | 2021.Q3 | 2021.Q4 |
| SOC1 |  |  | ↓ | ↓ | ↓ |  |  |  |
| SOC2 |  | ↓ |  |  |  |  |  |  |
| SOC3 |  | ↓ | ↓ |  |  |  |  |  |
| SOC4 |  | ↓ |  |  |  |  |  |  |
| SOC5 |  |  |  |  |  |  |  |  |
| SOC6 | ↑ | ↓ |  |  |  |  |  |  |
| SOC7 | ↑ | ↓ |  |  |  |  |  |  |
| SOC8 |  |  |  |  |  |  |  |  |
| SOC9 |  |  |  |  |  |  |  |  |

Table 3 Summary of findings from ARIMA models (based on full cohort) comparing expected and observed hypnotic rates

| ↓ = significantly lower than expected; ↑ = significantly higher than expected | | | | | | | | |
| --- | --- | --- | --- | --- | --- | --- | --- | --- |
|  | 2020.Q1 | 2020.Q2 | 2020.Q3 | 2020.Q4 | 2021.Q1 | 2021.Q2 | 2021.Q3 | 2021.Q4 |
| SOC1 |  |  |  |  |  |  |  |  |
| SOC2 |  |  |  |  |  |  |  |  |
| SOC3 |  |  |  |  |  |  |  |  |
| SOC4 |  | ↑ |  |  |  |  |  |  |
| SOC5 |  |  |  |  |  |  |  |  |
| SOC6 |  |  |  |  | ↑ |  |  |  |
| SOC7 |  |  |  |  |  |  |  |  |
| SOC8 |  |  |  |  |  |  |  |  |
| SOC9 |  |  |  |  |  |  |  |  |

Figure 2: Observed to expected ratios for antidepressants during the first 8 quarters of the COVID-19 pandemic (Quarter 1 2020 to Quarter 4 2021) among all NILS members in employment (includes fulltime and part-time workers) at the point of the 2011 Census.


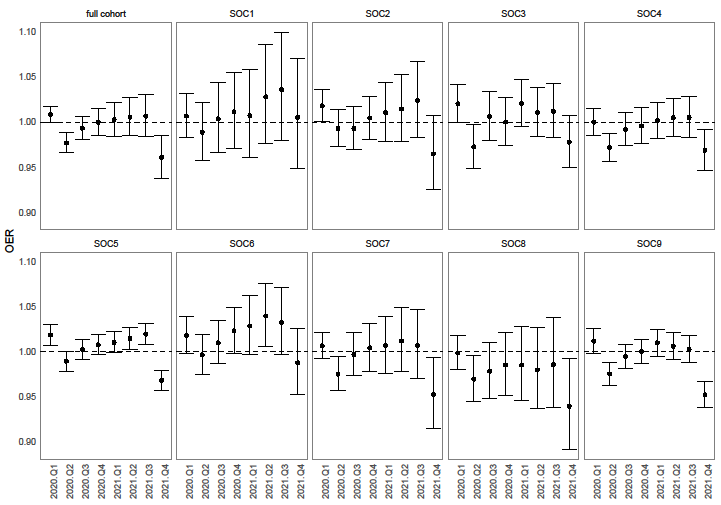


Figure 3 Observed to expected ratios for anxiolytics during the first 8 quarters of the COVID-19 pandemic (Quarter 1 2020 to Quarter 4 2021) among all NILS members in employment (includes fulltime and part-time workers) at the point of the 2011 Census.


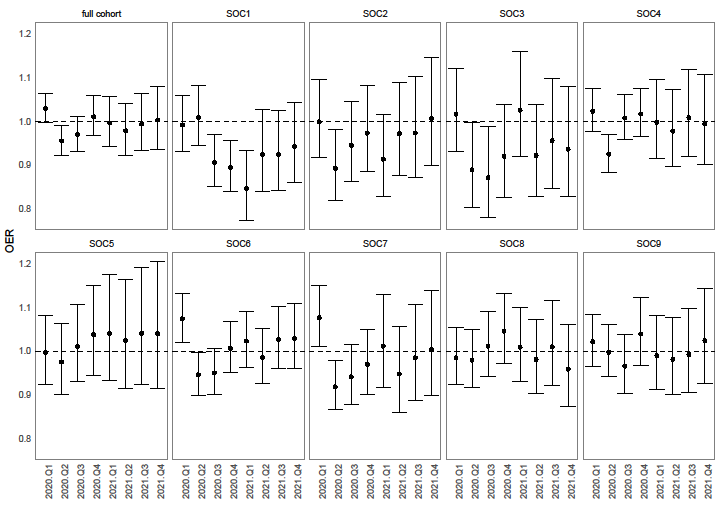


Figure 4 Observed to expected ratios for hypnotics during the first 8 quarters of the COVID-19 pandemic (Quarter 1 2020 to Quarter 4 2021) among all NILS members in employment (includes fulltime and part-time workers) at the point of the 2011 Census.


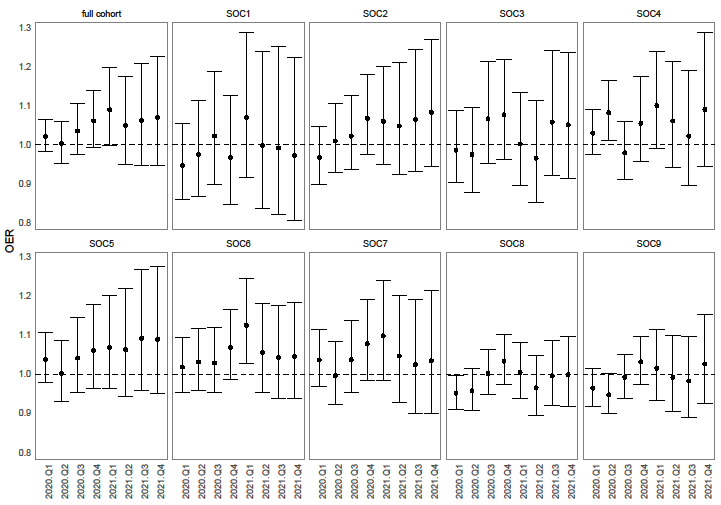


Sensitivity analyses based on ARIMA models that include full-time workers only (see Figures 5-8)

There is broad agreement between the two cohorts (main cohort including full-time and part-time workers (FT/PT cohort) and fulltime only cohort (FT cohort)) at population level.  There are however a few occupation groups showing differences between the main analysis and sub-analysis.

At full cohort level, each of the medications showed a lower rate of dispensation when we restrict to full-time workers.  This remains true across all occupation types for antidepressants and anxiolytics, and for most occupation types for hypnotics.  SOC6 (‘caring personal services’) sees an increased rate of hypnotics when we restrict to full-time workers and may reflect the strain of long and unsociable hours among full-time workers in these occupations during the pandemic and associated increase in sleep disorders.

Antidepressants

When we restrict to full-time workers only, we see that there is no longer a significant decrease in antidepressants in Q2 of 2020 for SOC4 (admin/sec), or a significant increase in Q2 and Q3 in 2021 in SOC5 (skilled trades). However, we do now see a significant increase in antidepressants in Q1, Q2 and Q3 of 2021 in full-time SOC7 (sales).  As alluded to in the main analysis, 2021 saw the substantial reopening of society; and the significant increase in antidepressants among this group of public facing sales workers may reflect worry associated increased exposure to Covid infection with return to the workplace on a FT basis.

Anxiolytics

When restricting to full-time only, we no longer see a significant decrease in anxiolytic use from Q3 in 2020 to Q1 in 2021 for SOC2 (managers, directors and senior officials), indicating the improvement in anxiety levels was largely accounted for by PT workers in these occupation types. In SOC6 (caring/leisure), we see a distinctly different trend in Q1 of 2020.  The FT/PT group saw an increase in anxiolytics, whereas there was a decrease in FT-only group.  This broad group includes leisure and travel occupations, teaching and childcare support, as well as caregiving occupations. The decrease in anxiolytics among FT workers may be associated with the alleviation of everyday working pressures among some of these groups in the wake of early restrictions.

Hypnotics

Full-time SOC5 (skilled trade) showed an increase in the use of hypnotics in late Q3 and Q4 of 2021, whereas this had failed to reach significance in the main analysis. In both the FT/PT and FT-only, there was an upward trend in hypnotic use, deviating from expected, and reaching significance in FT-only by the end of the study period, potentially reflecting the effects of adjustment to long working hours among FT time workers following the major reopening of society. The FT-only and FT/PT SOC6 (care/leisure) groups showed similar patterns in hypnotic use over the study period, both experiencing a peak in Q1 of 2021, however this was only significant in the main analysis.   This was also a time of "peaking" COVID-19 cases, and perhaps this also reflects concern amongst those PT workers with pre-existing conditions.

Figure 5 Auto regressive integrated moving average (ARIMA) illustrating forecast versus actual values of uptake of psychotropic medications during the first 8 quarters of the COVID-19 pandemic (Quarter 1 2020 to Quarter 4 2021) among all NILS members in full-time employment at the point of the 2011 Census. Dark grey band denotes the 80% confidence interval and the light grey band the 95% confidence interval


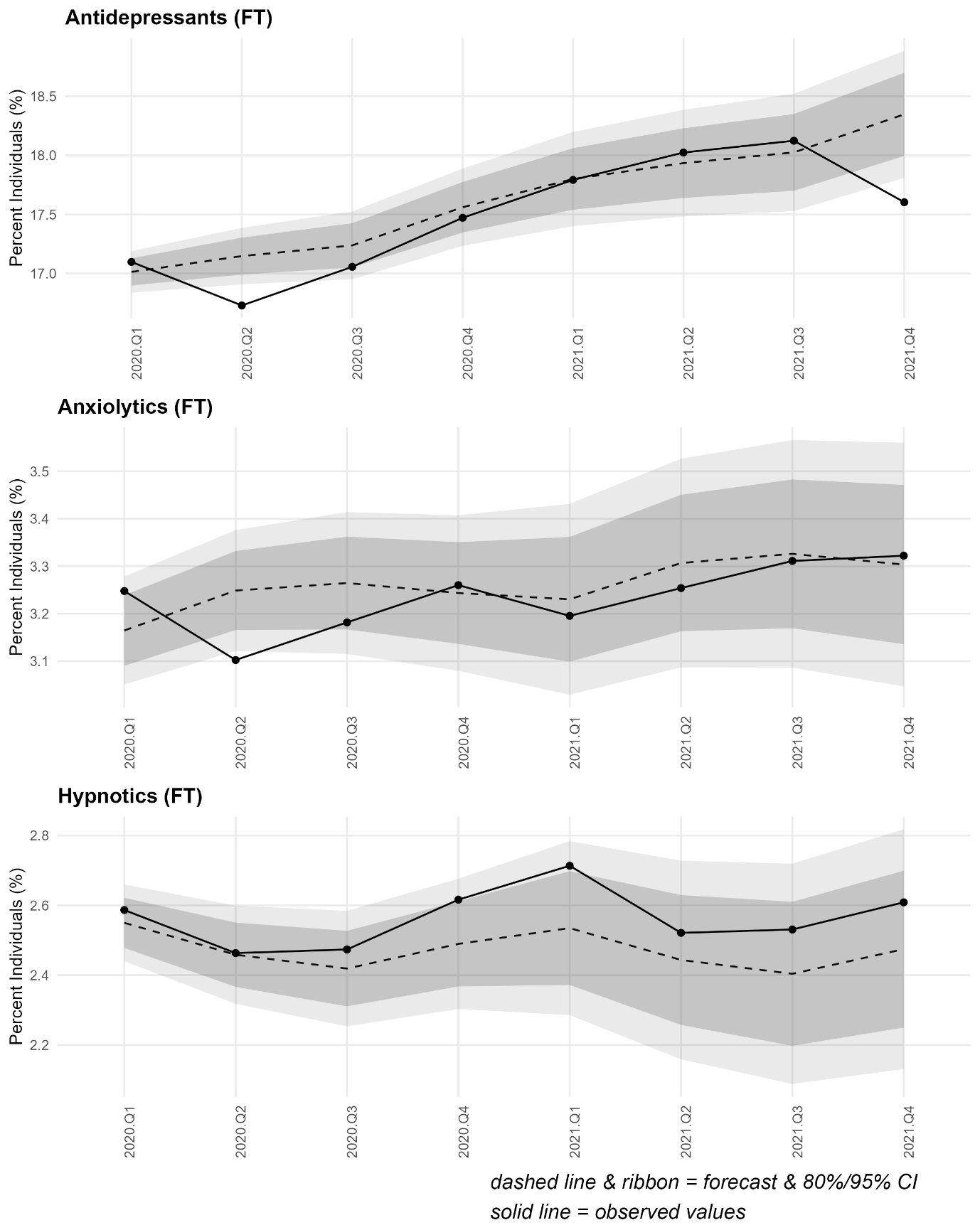


Figure 6 Auto regressive integrated moving average (ARIMA) illustrating forecast versus actual values of uptake of anti-depressant medications during the first 8 quarters of the COVID-19 pandemic (Quarter 1 2020 to Quarter 4 2021) among NILS members in full-time employment by standard occupational classification (SOC). Dark grey band denotes the 80% confidence interval and the light grey band the 95% confidence interval


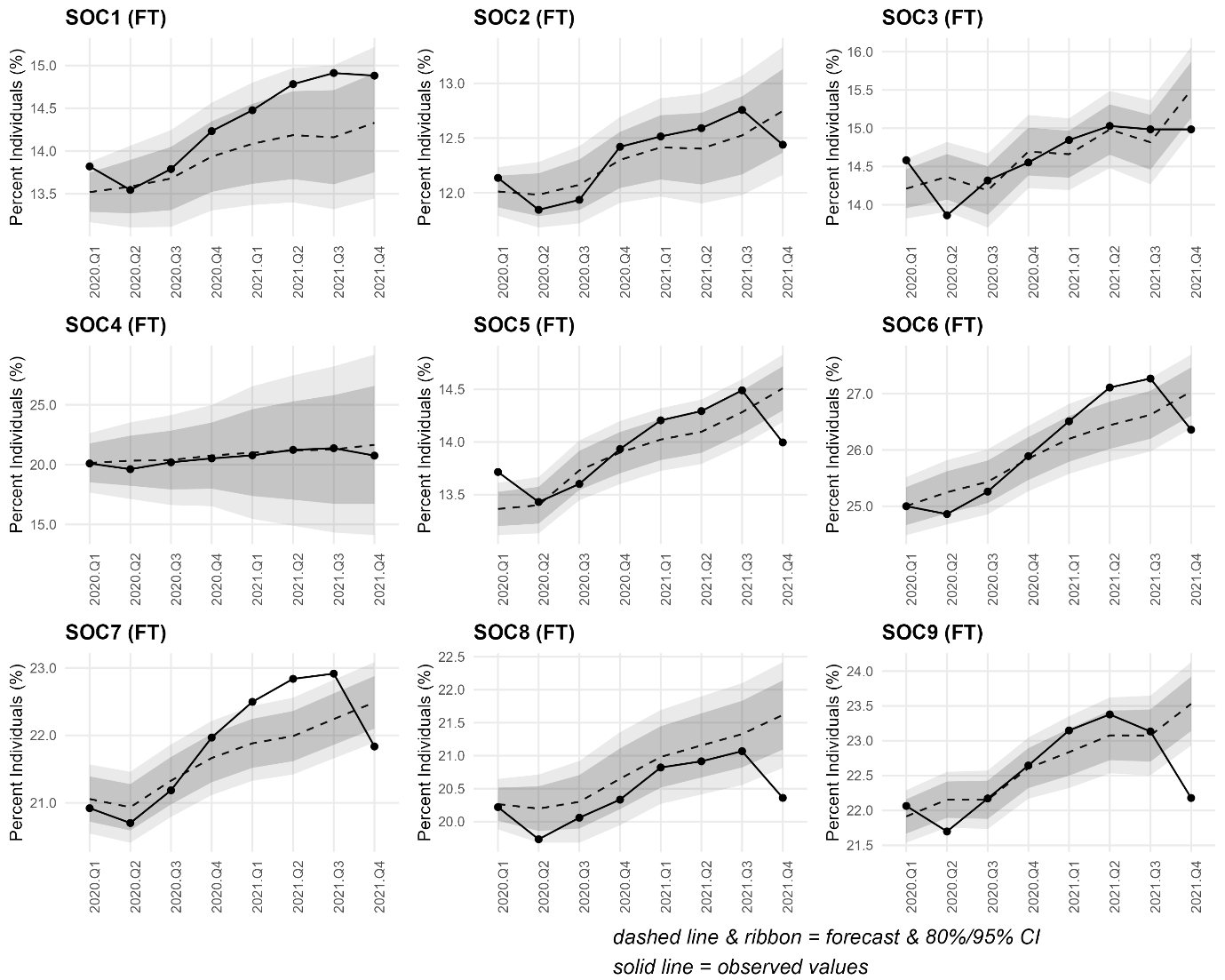


Figure 7 Auto regressive integrated moving average (ARIMA) illustrating forecast versus actual values of uptake of anxiolytic medications during the first 8 quarters of the COVID-19 pandemic (Quarter 1 2020 to Quarter 4 2021) among NILS members in full-time employment by standard occupational classification (SOC). Dark grey band denotes the 80% confidence interval and the light grey band the 95% confidence interval


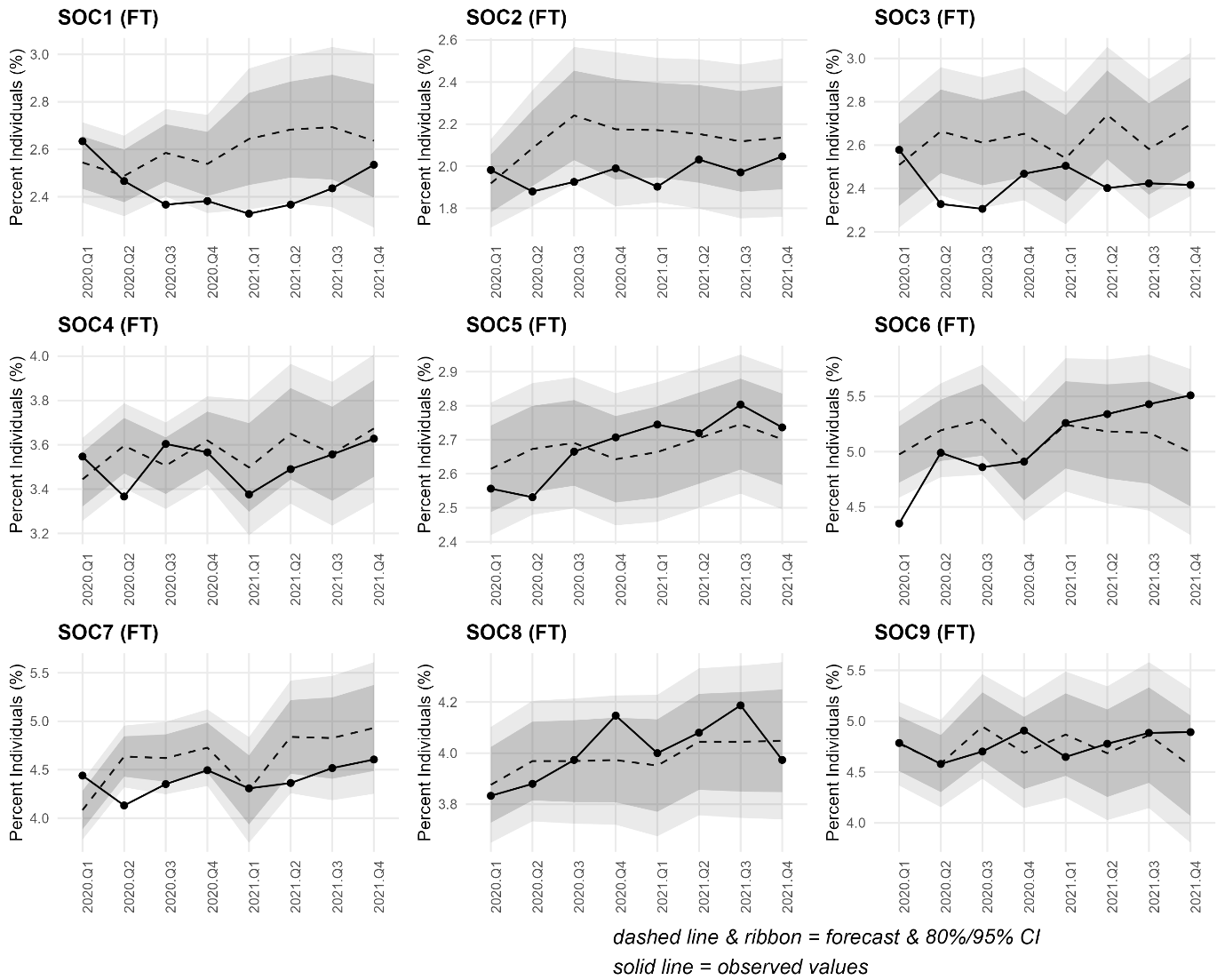


Figure 8 Auto regressive integrated moving average (ARIMA) illustrating forecast versus actual values of uptake of hypnotic medications during the first 8 quarters of the COVID-19 pandemic (Quarter 1 2020 to Quarter 4 2021) among NILS members in full-time employment by standard occupational classification (SOC). Dark grey band denotes the 80% confidence interval and the light grey band the 95% confidence interval


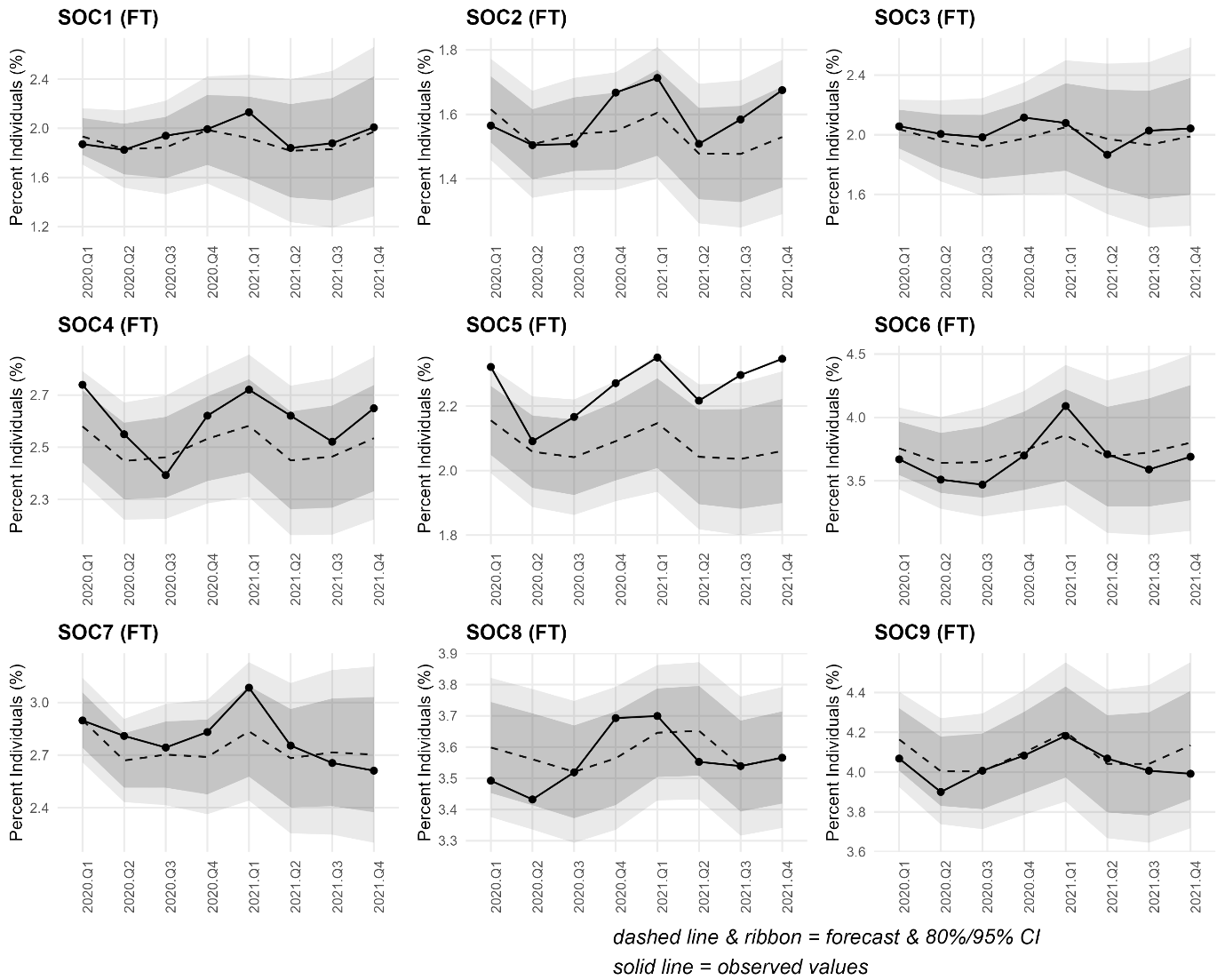

Supplement: Supplementary file 1 — Supplementary Material 1 [file 127_2025_2909_MOESM1_ESM.docx]
